# The Impact of Inotropes and Vasopressors on Cerebral Oxygenation in Patients with Traumatic Brain Injury and Subarachnoid Hemorrhage: A Narrative Review

**DOI:** 10.3390/brainsci14020117

**Published:** 2024-01-24

**Authors:** Michele Salvagno, Federico Geraldini, Giacomo Coppalini, Chiara Robba, Elisa Gouvea Bogossian, Filippo Annoni, Eva Vitali, Elda Diletta Sterchele, Costantino Balestra, Fabio Silvio Taccone

**Affiliations:** 1 Department of Intensive Care, Hôpital Universitaire de Bruxelles (HUB), 1070 Brussels, Belgium; 2 Department of Anesthesia and Intensive Care, Ospedale Università di Padova, 35128 Padova, Italy; 3 Department of Anesthesia and Intensive Care, Humanitas Clinical and Research Center, 20089 Milano, Italy; 4 Department of Biomedical Sciences, Humanitas University, Pieve Emanuele, 20072 Milano, Italy; 5 Anaesthesia and Intensive Care, IRCCS Policlinico San Martino, 16132 Genova, Italy; 6 Dipartimento di Scienze Chirurgiche Diagnostiche e Integrate, Università di Genova, 16132 Genova, Italy; 7 Department Environmental, Occupational, Aging (Integrative) Physiology Laboratory, Haute Ecole Bruxelles-Brabant (HE2B), 1160 Brussels, Belgium; 8 Anatomical Research and Clinical Studies, Vrije Universiteit Brussels (VUB), 1090 Brussels, Belgium; 9 DAN Europe Research Division (Roseto-Brussels), 1160 Brussels, Belgium; 10 Motor Sciences Department, Physical Activity Teaching Unit, Université Libre de Bruxelles (ULB), 1050 Brussels, Belgium

**Keywords:** vasopressors, inotropes, traumatic brain injury, subarachnoid hemorrhage, brain injuries, brain oxygenation, cerebral physiopathology

## Abstract

Traumatic brain injury (TBI) and subarachnoid hemorrhage (SAH) are critical neurological conditions that necessitate specialized care in the Intensive Care Unit (ICU). Managing cerebral perfusion pressure (CPP) and mean arterial pressure (MAP) is of primary importance in these patients. To maintain targeted MAP and CPP, vasopressors and/or inotropes are commonly used. However, their effects on cerebral oxygenation are not fully understood. The aim of this review is to provide an up-to date review regarding the current uses and pathophysiological issues related to the use of vasopressors and inotropes in TBI and SAH patients. According to our findings, despite achieving similar hemodynamic parameters and CPP, the effects of various vasopressors and inotropes on cerebral oxygenation, local CBF and metabolism are heterogeneous. Therefore, a more accurate understanding of the cerebral activity of these medications is crucial for optimizing patient management in the ICU setting.

## 1. Introduction

Brain function relies heavily on adequate tissue oxygenation and energy supply. Although the brain constitutes approximately 2% of the total body weight, it consumes roughly 20% of the total oxygen content [1]. This property renders the brain highly susceptible to any decrease in tissue oxygenation, with the risk of hypoxic brain damage and worse outcomes [2]. 

Traumatic brain injury (TBI) and subarachnoid hemorrhage (SAH) are two common neurological conditions that often require admission to the intensive care unit (ICU) and specialized monitoring and treatment [3]. Patients suffering from these brain injuries are at high risk for cerebral hypoxia, as a result of a combination of factors, such as vascular damage (with a reduction in cerebral blood flow, CBF), cerebral edema (which can elevate intracranial pressure, compressing blood vessels and further diminishing cerebral oxygenation) and systemic hypotension, anemia and/or hypoxemia [4,5]. As brain hypoxia has been associated with worse clinical outcomes, optimizing cerebral oxygenation is important in treating patients with brain injuries to prevent secondary brain damage [6].

Extracranial complications, particularly cardiac injuries, are frequently encountered in brain injury cases. For example, cardiac issues affect approximately 25–35% of patients with traumatic brain injury, ranging from changes in electrocardiogram readings to more serious conditions such as regional wall motion abnormalities, raised troponin levels and myocardial stunning [7]. Choosing inotropic and vasopressor medications for these patients requires careful consideration of these cardiac factors. Likewise, when employing these treatments to address the cardiovascular impacts associated with brain injuries, it is important to consider their effects on brain hemodynamics. Indeed, inotropes and vasopressors are frequently administered medications with the aim of maintaining cerebral perfusion pressure (CPP) within desired ranges, according to cerebral needs. Importantly, CPP, which is calculated as the difference between mean arterial pressure (MAP) and intracranial pressure (ICP; CPP = MAP − ICP), is one of the main determinants of CBF, together with cerebral vascular resistance (CVR; CBF = CPP/CVR) [8]. Inotropes and vasopressors can therefore influence cerebral perfusion by changing the driving pressure (i.e., CPP), or by increasing cardiac output and flow redistribution in the brain parenchyma or altering CVR [9]. Moreover, these drugs can also influence regional CBF and microcirculation, which directly regulate tissue oxygen delivery [10]. 

In animal models using healthy sheep, dopamine has demonstrated different impacts on ICP and cerebral oxygen utilization, compared to epinephrine and norepinephrine [11]. Similarly, in animal models of brain injury, norepinephrine and dopamine appeared to have differential effects on pericontusional cortical perfusion and glutamatergic transmission. Specifically, although both dopamine and norepinephrine substantially elevated MAP and CPP to comparable levels, norepinephrine led to a more marked enhancement in pericontusional cortical blood flow. This increase was sustained in the norepinephrine group for up to 30 min following infusion, while the dopamine group experienced a significant decline compared to pre-infusion levels. Additionally, in the animals treated with dopamine, extracellular levels of pericontusional glutamate remained unchanged, while in the ones receiving norepinephrine, there was a continuous rise in glutamate levels, peaking 30 min after the infusion was halted [12]. 

The existing literature is limited in identifying the most effective vasopressors and inotropes to be used in the management of TBI and SAH patients. Therefore, this manuscript aimed to partially fill this gap by reviewing current studies on how these medications impact cerebral oxygenation in these patients. 

## 2. Innervation and Regulation of Cerebral Vessels

**Figure 1:**
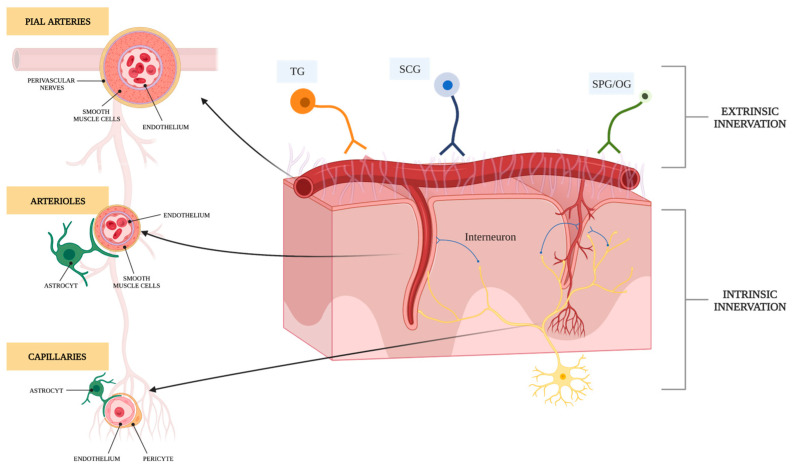
The sympathetic innervation of blood vessels in the brain. “Extrinsic” regulation involves nerves originating from peripheral ganglia (the superior cervical ganglion, sphenopalatine ganglion, otic ganglion and trigeminal ganglion) that surround the blood vessels outside the brain (pial arteries, which present multiple layers of vascular smooth muscle cells). However, once these nerves enter the brain tissue, the arterioles (small arteries of the brain’s surface, with a smaller layer of vascular smooth muscle cells) lose their nerve supply from the periphery. Instead, they and brain capillaries receive nerve fibers from central neuronal pathways (intrinsic innervation) and interneurons. Various cell types, including glia (astrocytes, microglia and oligodendrocytes), vascular cells (endothelial cells, vascular smooth muscle cells and pericytes) and neurons, are organized into well-structured and functionally integrated neurovascular units. These cellular components form a functional unit and blood–brain barrier (BBB) that maintains the brain’s microenvironment and detects and regulates cerebral blood flow (CBF) based on neuronal demand. This regulation occurs by converting neuronal signals into vasomotor responses, a process known as neurovascular coupling. SCG: superior cervical ganglion; TG: trigeminal ganglion; SPG: sphenopalatine ganglion; OG: otic ganglion.

Cerebral vessels are controlled by two distinct types of innervation, classified as “extrinsic” or “intrinsic” and based on the vessel’s position (Figure 1). Indeed, the parenchymal arterioles and cortical microvessels receive innervation from neurons within the brain tissue—such as dorsal raphe nucleus, locus coeruleus and fastigial nucleus (i.e., by an “intrinsic” innervation), while the pial arteries and arterioles up to the Virchow–Robin space are innervated by the peripheral nervous system (i.e., by an “extrinsic” innervation). This latter comprises sensory nerves (from the trigeminal ganglion), parasympathetic nerves (from the otic and sphenopalatine ganglia) and a sympathetic innervation (from the cervical ganglia) [13,14].

The sympathetic supply to cerebral vessels is derived from preganglionic fibers by neurons which rely on the upper thoracic segments, and then ascend along the sympathetic cervical chain and connect with postganglionic nerves in the cervical ganglia [13,15]. Nerve fibers emerge from the superior cervical ganglion to join the carotid nerve (which accompanies the internal carotid artery and its branches). In contrast, others from the stellate ganglion accompany the vertebral and basilar arteries and their branches up to the posterior cerebral arteries, where these join the innervation from the internal carotid artery [16,17,18]. Sympathetic innervations release adrenergic mediators that enhance sympathetic tone, a vital component of cerebral autoregulation. This regulatory process strictly controls CBF by responding to external factors (such as a drop in blood pressure or high carbon dioxide levels) and local factors (like transmural pressure or cerebral metabolism changes) [19].

**Figure 2:**
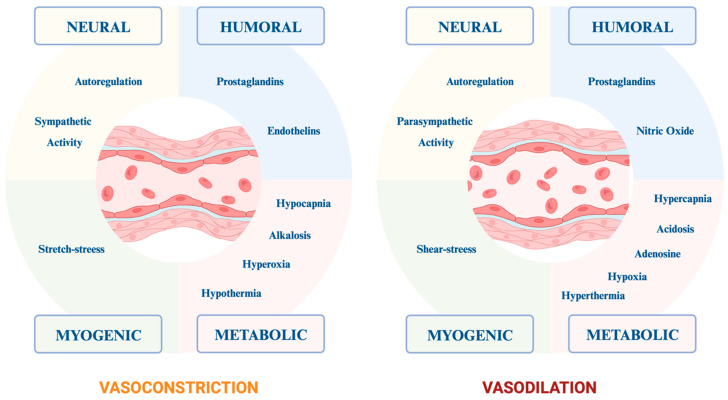
Regulatory mechanisms of cerebral vascular tone in a healthy brain. This figure delineates the principal elements that govern cerebral vascular tone, thereby modulating cerebral blood flow through vasodilation and vasoconstriction. The mechanisms are categorized into four primary groups: neural, humoral, myogenic and metabolic. The left panel highlights factors that induce vasoconstriction, such as reduced CO_2_ levels, hyperoxia, alkalosis and decreased temperature, along with mechanical stretch, prostaglandins, endothelins, autoregulatory processes and increased sympathetic activity. Conversely, the right panel elaborates on elements that promote vasodilation, including hypercapnia, hypoxia, acidosis, elevated temperature, shear stress, adenosine, prostaglandins, nitric oxide, parasympathetic activity and autoregulation.

The cerebral tissue is functionally isolated from the rest of the peripheral circulation by the multifunctional blood–brain barrier (BBB), composed of endothelial cells, astrocytes and pericytes. Together, these cell types form the neurovascular units [20], capable of monitoring and modulating CBF in response to neuronal activity by transducing neuronal signals into vasomotor responses. Along the course of the vessels, the main control of cerebral blood flow is largely governed by changes in vascular resistance in the brain’s parenchymal arterioles, with their substantial layer of smooth muscle cells and their significant capability for both dilation and constriction [21]. At this level, in a healthy brain, physiological factors influence this complex mechanism, as summarized in Figure 2. 

The impact of catecholamines on cerebral oxygenation should be read in the context of mechanisms that may vary between a healthy and an injured brain. First, the BBB serves as both a physical and enzymatic barrier for catecholamines, due to the presence of monoamine oxidases (MAO) and other enzymes [22,23] whose activity may be, therefore, locally diminished under physiological conditions. In the setting of brain injuries, the alteration of the BBB may alter the expression of these enzymes, resulting in the heightened activity of catecholamines due to the lack of counterbalance. Moreover, while it is generally understood that catecholamines typically cannot easily penetrate the BBB to enter the brain, except at circumventricular sites where the BBB is absent or less effective [23], experimental evidence suggests paradoxical effects of vasopressors in the context of an acute brain injury, including increases in cerebral blood flow and the cerebral metabolic rate of oxygen, in the setting of BBB disruption [24,25,26]. Similarly, autonomic dysfunction, in particular of the parasympathetic system, which can develop after TBI and SAH, could impair the system’s ability to mitigate cerebral vessel vasoconstriction [13,18]. Other factors, such as the potential concurrent use of sedatives and anesthetics in TBI and SAH patients, could alter cerebral vasoreactivity. For example, thiopental resulted in reduced CBF and cerebral metabolic oxygen consumption (CMRO_2_), blunting vasomotor reactivity while preserving cerebral autoregulation [27,28]. Such effects could potentially interfere with the impact of vasopressors on cerebral vessels. It has also been shown that catecholamines stimulate the cerebral metabolism without changes in cerebral hemodynamics. Indeed, as is known, cerebral energy metabolism predominantly relies on glucose oxidation, as evidenced by the ratio of cerebral oxygen uptake to carbohydrate and lactate uptake. This ratio [cerebral molar uptake ratio O_2_/(glucose + (1/2) lactate)] is known as the Oxygen–Carbohydrate Index (OCI) or the Cerebral Metabolic Ratio (CMR), and its normal value is approximately 6 [29,30]. Adrenaline, but not noradrenaline, has been shown to lower the OCI value, without a significant change in cerebral blood flow velocities, supporting that non-oxidative carbohydrate consumption for the brain is driven by a β_2_-adrenergic mechanism [29]. Lastly, vasopressor medications may also affect the functioning of the aquaporin system, a group of water channel proteins identified as a possible contributing factor in both the development and resolution of edema in the setting of brain injuries [31]. Indeed, aquaporin-4, a member of this family, holds particular significance in the brain due to its location in perivascular areas. Vasopressor drugs, such as dopamine, have been demonstrated to interact with aquaporin-4, influencing either its water permeability or expression levels [32,33], and therefore influencing tissue oxygen diffusion.

## 3. Adrenergic Receptors

Adrenergic receptors, also known as adrenoceptors, are a class of G-protein-coupled receptors targeted by catecholamines. Among other physiological responses, they may also cause vasodilation or vasoconstriction and therefore modulate vascular diameter. Adrenoceptors are classified into two main types, alpha (α) and beta (β) receptors, further subdivided into α_2_, α_2_, and β_1_, β_2_, β_3_. Each subtype has distinct physiological roles, signaling pathways and distribution patterns [34]. 

Generally, vasoconstrictive responses are attributed to α1-receptors, representing the most frequent receptor in cerebral circulation. The binding to the receptors causes the activation of GTPase hydrolase enzymes called G_q_ proteins, triggering phospholipase-C activation which, in turn, results in the production of inositol triphosphate (IP3) and diacylglycerol (DAG), thereby raising the levels of calcium inside the cell [35]. α_2_-receptors in the pre-synaptic cleft inhibit the release of norepinephrine, thereby providing a negative feedback-loop mechanism for neurotransmission. Post-synaptic α_2_-receptors, like α_1_-receptors, promote vasoconstriction [36,37]. 

β_1_ receptors predominantly influence cardiac activity, increasing heart rate and contractility [38]. Along the cerebral arteries, with a β_1_-β_2_ ratio of 4:6, β-receptors are mainly located in the post- and pre-synaptic cleft and are typically involved in smooth muscle relaxation and, thus, the dilation of blood vessels [39]. The β_3_ receptors, though less well characterized, are believed to be involved in fat metabolism.

The cerebrovascular system, which includes arteries and arterioles that supply the brain, possesses all these subtypes of adrenergic receptors, all of which play crucial roles in regulating cerebral blood flow. In the cerebrovascular endothelium, β2-adrenergic receptors are the most abundant. Their activation leads to the production of nitric oxide (NO), a potent vasodilator, thus promoting an increase in cerebral blood flow [40]. Conversely, α1-adrenergic receptors are predominantly located on the smooth muscle cells of cerebral blood vessels, and they are the most diffuse adrenergic receptors in the brain vasculature. Their activation leads to vasoconstriction and potentially decreases CBF [41]. α_2_-adrenergic receptors are also present in the cerebrovascular system, but their exact distribution and physiological role in this context are not as well defined as α_1_ and β_2_ receptors. Nonetheless, they are additionally linked to vasodilation mediated by endothelial cells.

**Figure 3:**
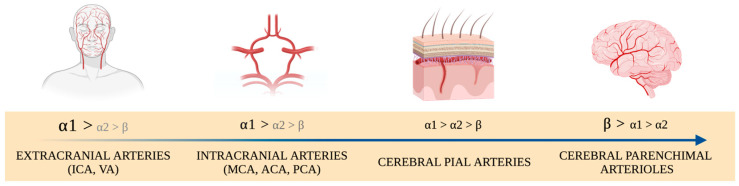
The distribution of the adrenoceptors along the cerebral vasculature. Adapted from Brassard et al. [42]. Pial arteries run along the brain’s surface, within the pia mater layer of the meninges. Parenchyma arteries are situated within the brain tissue and are generally smaller in diameter compared to pial arteries. They are the terminal branches of pial arteries and are primarily responsible for supplying blood directly to the brain tissue. Due to their deeper location within the brain, they are more involved in forming the blood–brain barrier and more regulated by local factors within the brain tissue, such as pH and CO_2_ levels. MCA: Middle Cerebral Artery; ICA: Internal Carotid Artery; VA: Vertebral Artery; ACA: Anterior Cerebral Artery; PCA: Posterior Cerebral Artery.

Starting from the major extra-cranial arteries, there is a decrease in the responsiveness to α-adrenergic receptors, accompanied by a gradual transition to β-adrenoceptors, moving toward the intracranial arteries and the small pial arterioles, eventually becoming nearly absent in parenchymal arterioles [42] (Figure 3). 

Dopamine receptors are a class of G-protein-coupled receptors (but not counted as adrenergic ones) primarily targeted by dopamine. Five subtypes of dopamine receptors have been identified and are categorized into two major families: the D1-like family (which includes the D1 and D5 subtypes that stimulate the production of cyclic adenosine monophosphate (cAMP) by activating the enzyme adenylyl cyclase via Gs proteins) and the D2-like family (which includes the D2, D3 and D4 subtypes that inhibit the production of cAMP). D1-like receptors have been shown to be present and mediate vasoactive effects at the level of the pial cerebral vessels [43,44]. The significance of these receptors in the cerebral circulatory changes caused by the systemic administration of dopamine has not been clearly defined. D2-like receptors, particularly D2 receptors, are also present in the cerebral vasculature, but their precise role in cerebrovascular regulation is less clear.

Different drugs cause the differential activation or inhibition of these receptor subtypes due to their varying affinities [45]. For example, epinephrine demonstrates a high affinity for α1, β1 and β2 receptors, with the greatest affinity for α1 receptors. Norepinephrine, similar to epinephrine, shows a strong affinity for α1 and β1 receptors. Indeed, it is a potent vasopressor, ideal for managing hypotension. Dopamine presents a unique receptor profile with significant affinity for dopamine receptors, in addition to moderate affinity for β1. Dobutamine is characterized by its high affinity for β1 receptors and moderate affinity for α1 and β2 receptors, with a role in managing heart failure and cardiogenic shock. Phenylephrine is a selective α1 agonist, useful in treating hypotension without significantly affecting heart rate. Finally, isoproterenol shows exclusive affinity for β1 and β2 receptors, with chronotropic effects. These differences can also lead to different effects on cerebrovascular dynamics and may have implications for conditions such as stroke, traumatic brain injury and other cerebrovascular disorders. 

Indeed, in a healthy brain, the impact of vasopressor agents on the central nervous system is modulated by multiple factors that contribute to the regulation of cerebrovascular tone. This complexity may be further amplified in the setting of brain injury, as previously described (see Figure 2).

In animal models where brain injury was induced through experimental fluid percussion, the concentration of dopamine and norepinephrine in brain tissues was significantly higher than in control samples, starting from a few hours after the trauma until two weeks after the induced injury [46]. This indicates that not only can brain permeability to catecholamines change after trauma, but acute alterations in regional concentrations of brain catecholamines following brain injury may persist for extended periods. In the case of SAH, it has been observed that the concentration of β1 adrenergic receptors increases and this, combined with vasodilation, could potentially serve as a protective response to mitigate vasospasm [39].

Although research has investigated the intracerebral effects of vasopressors in animal models with BBB alterations, there is limited clinical evidence regarding the different cerebrovascular effects of vasopressors and their concentrations within brain tissue when the BBB is compromised. For example, TBI patients reported a paradoxical reduction in regional cerebral blood flow after the norepinephrine-induced elevation of cerebral perfusion pressure [47]. This reduction in blood flow was hypothesized to be a consequence of increased BBB permeability, allowing norepinephrine to diffuse across endothelial cells and directly constrict cerebral vessels. Similarly, norepinephrine was associated with the vasoconstriction of cerebral vessels in an animal model with induced BBB disruption, leading to speculation that the BBB impedes a potential α-adrenoceptor-mediated vasoconstrictive effect of norepinephrine [48].

## 4. Cerebral Oxygenation Measurements

There are various methods to evaluate cerebral oxygenation, with three being the most frequently utilized, either through direct or indirect assessment [49]. First, a direct measurement involves inserting an oxygen electrode into the brain tissue, a method known as brain tissue partial oxygen pressure (PbtO_2_). This technology provides continuous, localized measurements of brain tissue oxygenation; however, it is also invasive and carries risks such as infection or hemorrhage, and the data are confined to the area around the probe. In contrast, jugular venous oxygen saturation (SjvO_2_) monitoring entails placing a catheter in the jugular vein to gauge the oxygen saturation in blood flowing out of the brain. This technique offers an indirect assessment of cerebral oxygenation and metabolism, but it might not capture regional variations, as it only provides a global view. Finally, Near-Infrared Spectroscopy (NIRS) is a non-invasive method using infrared light to measure cerebral oxygenation. NIRS estimates the oxygenation of hemoglobin in the brain’s superficial vessels, but its scope is restricted to cortical tissue, and several factors like skin perfusion and thickness can affect its accuracy.

Moreover, other approaches can be used. The arterio-venous difference of oxygen (AVDO_2_), achieved by simultaneously sampling arterial and jugular venous blood, shows the oxygen content difference, giving an estimate of cerebral oxygen consumption [50]. Similarly, the Oxygen Extraction Fraction (OEF) denotes the proportion of oxygen that is extracted from the blood during its passage through the brain [51]. Lastly, the calculation of the Cerebral Metabolic Rate of Oxygen (CMRO_2_) typically involves analyzing cerebral blood flow (CBF) alongside AVDO_2_ [52]. This measurement serves as an indicator of the brain rate of oxygen consumption; however, its use is less relevant in clinical practice, as the values are not continuous.

## 5. Effects of Adrenergic Inotropes and Vasopressors on Cerebral Oxygenation

### 5.1. Dopamine

**Figure 4:**
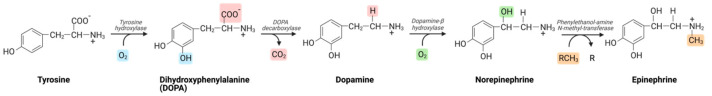
Catecholamine neurotransmitter synthetic pathway. Exogenous dopamine administered therapeutically has many effects, depending on its action on α, β, DA (dopamine) and 5-hydroxytryptamine (5-HT) receptors. Its action also depends on the administered dose, commonly categorized into low (0.5 to 3 μg/kg·min), moderate (3 to 10 μg/kg·min) and high doses (10 to 20 μg/kg·min), evoking responses on different receptors.

Catecholamines (Dopamine, Norepinephrine, Epinephrine) are organic compounds derived from tyrosine, consisting of a benzene ring with two hydroxyl side groups adjacent to each other and a side-chain amine. Dopamine is a natural catecholamine, the first in the catecholamine synthetic pathway (see Figure 4), an immediate precursor to norepinephrine and an endogenous central neurotransmitter. 

Low doses act primarily on dopaminergic D_1_ postsynaptic receptors and D_2_ presynaptic receptors (concentrated in the coronary, renal, mesenteric, cerebral bed, vasculature and renal tissues), ultimately promoting vasodilation and increased blood flow. At moderate doses, the effect on β_1_-adrenergic receptors is dominant, with action on presynaptic sympathetic nerve terminals, which results in increased cardiac contractility and chronotropic activity, while only having a small effect on systemic vascular resistance. The effect is primarily of vasoconstriction at high doses, mediated by the α_1_-adrenergic receptor [45].

Clinical uses of dopamine have been declining, especially as low doses, sometimes referred to as “renal doses”, have not been showing any significant benefits to renal function [53]. In cases of shock, it is frequently replaced by alternative vasopressors. Its use remains typically in addition to other vasopressors or when first-line vasopressors are unavailable [54,55,56].

#### Cerebral Oxygenation Effects

The effects of dopamine on the cerebral vascular system are not yet completely understood. Dopamine appears to have vasoconstrictive effects on major cerebral arteries, resulting from its activity on α_1_ and 5-HT receptors. Studies indicate that dopamine may increase CBF, albeit less predictably than the more commonly utilized norepinephrine [57]. Notably, dopamine may elicit a greater elevation in ICP at equivalent MAP compared to norepinephrine. However, in these investigations, CBF is relatively unaffected, likely due to intact autoregulation mechanisms [58]. Nevertheless, the effects of dopamine on cerebral oxygenation are variable. Indeed, CMRO_2_ seems generally unchanged by dopamine infusion. By contrast, when the BBB is damaged and dopamine can easily cross it, an increase in CMRO_2_ has also been detected [59].

In their research, Kiening et al. [60] examined individuals who had a Glasgow Coma Scale (GCS) score of 8, after experiencing TBI or intracerebral hemorrhage (ICH). Regardless of the ICP level, they aimed to maintain a CPP above 60 mmHg by administering isotonic fluids and dopamine. In one group, the CPP was increased each time it dropped below 40 mmHg (from an average of 32 ± 2 to 67 ± 4 mmHg). This led to a significant improvement in brain tissue oxygenation (increasing from 13 ± 2 to 19 ± 3 mmHg), and jugular venous oxygen saturation (increasing from 54 ± 3 to 65 ± 3%). Additionally, a decrease in ICP was observed, indicating that cerebral autoregulation was functioning correctly. However, in another group, maneuvers elevating the CPP even further in patients with a normal CPP (>60 mmHg), from 68 ± 2 to 84 ± 2 mmHg did not produce any significant changes in SjvO_2_, brain oxygenation or ICP. Thus, dopamine, with its effects on vascular and systemic factors, presumably may help to maintain appropriate oxygenation. Nevertheless, this effect diminishes when CPP is adequate. 

A study by Unterberg et al. [61] examined 21 patients with TBI during various treatment modalities, such as dopamine and mannitol infusion, and their effect on several parameters including tissue brain oxygenation and CPP. Dopamine administration significantly increased brain oxygenation, as indicated by increased PbtO_2_ and SjvO_2_ levels, along with an elevation of CPP from an average of 32 ± 2 to 67 ± 4. However, further increases in CPP (from 68 ± 2 to 84 ± 2) did not show any additional effects on brain oxygenation parameters. On the other hand, 125 mL of 20% mannitol administration did not lead to any significant changes in PbtO_2_ or SjvO_2_, despite a significant increase in CPP in patients with an ICP baseline level >20 mmHg. This might suggest that dopamine has an additional impact on oxygenation beyond that resulting from increased CPP. However, among patients administered mannitol, the CPP baseline was already over 60 mmHg, which could explain or contribute to this lack of effect observed.

### 5.2. Norepinephrine

Norepinephrine, produced by the β-oxidation of dopamine, is a potent vasoactive drug that remains the first choice to support arterial pressure in septic shock situations [62]. Its extensive use is derived from its pharmacological properties, as it acts on α_1_ and ß_1_ receptors with a resulting increase in venous and arterial tone, preload and both chronotropic and inotropic effects, acting as a mild stimulator of β-adrenergic receptors and a strong activator of α-adrenergic receptors. In addition to general ICU, norepinephrine is widely used in the neuro-ICU to maintain adequate CPP. Still, its impact on the cerebrovascular system remains unclear, with conflicting results regarding its influence on CBF changes [63].

#### Cerebral Oxygenation Effects

Norepinephrine is one of the most used drugs in TBI to maintain an adequate CPP. When compared to dopamine, evidence has suggested that norepinephrine may improve global and regional brain oxygenation [64]. Nevertheless, continuous noradrenaline infusion has also been linked to a reduction in cerebral tissue oxygen saturation (SctO_2_) in both anesthetized [65] and healthy patients [66]. In both of these studies, the lack of elevation in cardiac output (CO), the main determinant for CBF, may account for the observed absence of enhanced cerebral oxygenation, despite the elevated MAP. 

In a study evaluating the effect of hypertension in TBI [67], ^15^O-PET undertaken on 10 healthy volunteers as controls was compared to 20 TBI patients with a median GCS 7 and a CPP target of ~70 mmHg, achieved with norepinephrine with a mean range of 0.07 (0–0.25) µg/kg/min. In the TBI group, PET was repeated after increased CPP, to achieve a target of ~90 mmHg with an infusion of norepinephrine with a mean range of 0.15 (0.05–0.33) µg/kg/min. Compared to control, TBI patients at baseline had similar CBF, but a lower global CMRO_2_ and oxygen extraction fraction (OEF) and higher CBV. After that, the augmentation of CPP from ~70 to ~90 mmHg resulted, in all patients, in a significant increase in CBF and a reduction in OEF and CMRO_2_. Interestingly, the authors observed that decreases in CMRO_2_ were observed in the brain regions where OEF reduced the most. This suggests that the noted decrease in oxygen extraction could be a result of an increase in oxygen delivery, which is a predictable outcome of elevated CPP due to norepinephrine administration, and a reduction in oxygen demand, evidenced by the reduced CMRO_2_, which is unexpected, and not easy to explain. 

In an elegant study, Johnston et al. [64] compared the effects of dopamine and norepinephrine when raising CPP by 20 mmHg from a ~70 mmHg baseline in 11 TBI patients. As expected, they found major differences in the hemodynamics effect, with dopamine showing greater changes in cardiac index, and norepinephrine showing a greater effect on the systemic vascular resistance index. Despite these systemic hemodynamic differences between dopamine and norepinephrine, there were no significant disparities in regional and global cerebral oxygenation, as measured by PbtO_2_ and arterial–venous differences in oxygen (AVDO_2_), at either level of CPP. However, as previously reported, it was observed that dopamine had a much more pronounced and variable impact on AVDO_2_ between the baseline and the increase (3 ± 26%), compared to norepinephrine (−11 ± 8%). Additionally, dopamine led to a significant elevation in lactate and pyruvate levels. Overall, the response of AVDO_2_ to the CPP intervention with dopamine displayed greater interpatient variability than the response achieved with norepinephrine. This difference may be related to the intricate pharmacokinetics and pharmacodynamics of dopamine in critically ill patients, in contrast to norepinephrine, which exhibits more predictable pharmacokinetics, consistent with single-compartment first-order kinetics [68].

Successively, Johnston’s group [69] evaluated whether a CPP augmentation (from ~70 mmHg to ~90 mmHg) with norepinephrine may result in any improvement in cerebral metabolic function, evaluated by ^15^O-PET, PbtO_2_ and microdialysis in a group of 11 TBI patients. The study revealed a significant increase of 5 mmHg in PbtO_2_ (from 17 ± 8, to 22 ± 8 mmHg), which could not be attributed to an increase in PaO_2_, and a significant reduction in oxygen extraction fraction. However, despite a significant increase in CBF, no correlation was observed between CBF and PbtO_2_. Similar to the preceding study’s findings, no significant alterations in brain metabolism were detected when studied by microdialysis variables.

Recently, PbtO_2_ was utilized to measure tissue oxygenation during CPP adjustments in a study conducted by Kovacs et al. [70], which aimed to assess the CPP threshold that correlates with sufficient PbtO_2_ levels in brain-injured patients (the target was >20 mmHg). In total, 53 patients with heterogeneous types of ABI (TBI, SAH or ICH) received an infusion of norepinephrine to reach increases in CPP of at least 10 mmHg, 10–15 min each, at two different steps above the baseline, from 73 (70–76) to 83 (80–86) and to 92 (90–96), with a progressive PbtO_2_ increase from 20 (17–23) to 22 (20–24) and 24 (22–26), respectively. Focusing on CPP, they concluded that PbtO_2_ monitoring indicated the need for higher CPP targets than recommended to prevent tissue hypoxia.

Lang [71] manipulated arterial pressure with norepinephrine to achieve CPP ranging from 50 to 100 mmHg in 14 severe TBI patients. In patients with more intact autoregulation, the overall changes in PbtO_2_ were less pronounced when CPP increased. This finding establishes a significant correlation between cerebral autoregulation and cerebral tissue oxygen reactivity. In other words, the better the autoregulation is preserved, the smaller the increase in PbtO_2_ in response to CPP changes. Furthermore, the correlation between PbtO_2_ and the CPP they investigated is captivating. Indeed, in most cases, an increase in ABP causes an increase in PbtO_2_. However, it exhibits an ellipsoidal pattern caused by a time lag of 20 s to 3 min in PbtO_2_ changes following an increase in arterial blood pressure. This observation paves the way for further speculative considerations, discussed later.

### 5.3. Phenylephrine and Ephedrine

Phenylephrine is a medication that acts as a selective α_1_ agonist. Its use to improve CBF and brain oxygenation is a topic of debate. While phenylephrine can increase vascular resistance and thus raise CPP, there is insufficient evidence to definitively support its clinical advantages in elective neuroanesthesia and brain-injured patients [72,73]. Indeed, as it may cause reflex bradycardia, a reduction in CO may be elicited.

The presence of an N-methyl group in the ephedrine molecule decreases binding affinities at α receptors, compared with norephedrine. Its stimulatory effect on adrenergic receptors is both direct and indirect. Due to its rapid action, it is extensively utilized to swiftly augment MAP during hypotensive events, such as those occurring during the induction of anesthesia.

#### Effects on Cerebral Oxygenation

Studies have shown that phenylephrine can increase blood velocity in the middle cerebral artery (MCA), possibly due to vasoconstriction, although blood flow in the internal carotid artery remains unchanged. In a randomized trial involving brain tumor patients, the changes in CMRO_2_ were similar between those who received phenylephrine and those who received ephedrine. Both phenylephrine and ephedrine increased CBF and brain tissue oxygenation in the tumor area, but ephedrine had a more pronounced effect. Only ephedrine increased CBF and brain tissue oxygenation in the unaffected contralateral region.

In a study performed by Pedersen et al. [74], they evaluated the effects of an intravenous bolus of phenylephrine (0.1–0.2 mg) in changing the PbtO_2_ and ScvO_2_ measures from 5 min before to 5 min after the bolus in patients undergoing craniotomy under general anesthesia. Despite the increase in arterial blood pressure, the brain tissue oxygen tension remained unchanged.

### 5.4. Dobutamine

Dobutamine is a synthetic catecholamine invented in 1975 and originally designed as an inotropic agent for use in congestive heart failure [75]. Due to its molecular structure, with an asymmetric carbon atom, dobutamine has two different enantiomers with different affinities for adrenergic receptors [76]. Experimental studies on laboratory rats [77,78] have shown that the negative isomer (−)-dobutamine is primarily an α_1_-receptor agonist and produces marked increases in cardiac output, stroke volume, total peripheral resistance and mean arterial pressure, but does not significantly increase heart rate. Conversely, the positive isomer (+)-dobutamine is a β_1_ and β_2_-receptor agonist and elicits only a modest increase in cardiac output, due almost entirely to increased heart rate, and decreases SVR and MAP. Additionally, dobutamine is rapidly metabolized into (1)-3-O-methyl-dobutamine, which exerts α_1_ inhibitory effects and decreases myocardial contractility and SVR [78]. Since dobutamine pharmacological preparations consist of 50% (−)-dobutamine and 50% (+)-dobutamine, the net effects of the drug administration are increases in myocardial contractility and, to a lesser extent, heart rate, and either no effect or a decrease in SVR. 

Dobutamine is used in various clinical settings, from the cardio lab in performing pharmacological stress echocardiography to ICUs for treating sepsis-associated cardiovascular failure. In neuro-ICUs, dobutamine has been used to increase blood pressure and grant adequate CPP in cases of neurogenic stunned myocardium, a well-described complication of acute neurological injuries such as SAH or TBI [79].

#### Cerebral Oxygenation Effects

The effects of dobutamine on brain oxygenation remain unknown, and only a few studies have explored this topic. Recently, Coppalini et al. conducted a retrospective study on patients with acute brain injury to examine the potential impact of inotropes on cerebral oxygenation [9]. The study included 35 patients, 34 receiving dobutamine and 1 receiving milrinone. Results showed that approximately 65% of the patients experienced an improvement in brain tissue oxygenation, although only half demonstrated a significant increase in PbtO_2_ (defined as >20% from the baseline). Baseline tissue hypoxia was found to be the most reliable predictor of PbtO_2_ response in these patients, with lower levels of baseline PbtO_2_ being associated with a greater increase after administering inotropes. Interestingly, among the patients identified as “responders”, there was a direct correlation between changes in PbtO_2_ and cardiac output and a negative correlation between changes in PbtO_2_ and CPP. These observations support the hypothesis of “cardio-cerebral coupling”, where inotropes improve brain hemodynamics and oxygenation. The optimization of global hemodynamics had a positive impact on cerebral oxygenation. The observed decrease in CPP, likely achieved through vasodilation mediated by β_2_ receptors, did not have a detrimental effect on cerebral hemodynamics. This suggests the importance of monitoring systemic hemodynamics to fully understand the effects of increasing cardiac output on brain oxygenation. These discoveries emphasize the importance of adopting a “hyperdynamic” strategy, which prioritizes enhancing overall systemic hemodynamics and comprehending additional mechanisms like the direct impact on cerebral vessels. This approach goes beyond raising blood pressure to manipulate cerebral hemodynamics in patients with brain injuries, aiming to enhance brain oxygenation.

These findings confirm a precedent prospective study in which dobutamine was used to induce a rapid and transient increase in cardiac output, followed by a stabilization of cerebral oxygenation (measured by regional cerebral oxygen saturation), in patients with symptomatic vasospasm following SAH [80]. Dobutamine therapy effectively reversed cerebral ischemia, and they found a strong correlation between the peak slope of the artery-based pulse contour cardiac output and regional cerebral oxygen saturation.

### 5.5. Phosphodiesterase Inhibitors

Phosphodiesterase inhibitors are used in various clinical settings, including chronic obstructive pulmonary disease, erectile dysfunction, pulmonary arterial hypertension, benign prostatic hyperplasia, acute decompensated heart failure, psoriasis, psoriatic arthritis, atopic dermatitis and neonatal apnea [81]. A group of these medications, which include milrinone and enoximone, act as inhibitors of the enzyme’s phosphodiesterase 3 (PDE-3) and are primarily used to treat decompensated cardiac failure and peripheral arterial disease. However, they have occasionally been used in patients with SAH and TBI [82,83,84,85,86]. They exert their effects by preventing the degradation of cyclic guanosine monophosphate (cAMP), leading to an increase in these substances in smooth muscle cells. This increase results in relaxation and vasodilation in both the myocardium and peripheral vasculature. Additionally, these inhibitors cause a positive inotropic effect by increasing ionized calcium in the myocardium and reducing platelet aggregation. As nonspecific inhibitors (alongside PDE-4 and 5), they induce bronchial relaxation and help decrease pro-inflammatory mediators [81].

#### Cerebral Oxygenation Effects

Several studies have reported a vasodilatory effect on cerebral arteries and arterioles, leading to increased CBF [85,87,88,89]. The vasodilatory properties of PDE-3 inhibitors have also been demonstrated in several studies involving patients with SAH, showing the potential to attenuate cerebral vasospasm [90,91,92]. Nevertheless, there is still a lack of extensive research on the impact of PDE-3 inhibitors on cerebral oxygenation.

## 6. Effects of Non-Adrenergic Inotropes and Vasopressors on Cerebral Oxygenation

### 6.1. Levosimendan

Levosimendan, initially introduced for treating acute heart failure, is a medication that acts as a positive inotropic drug with vasodilator properties by sensitizing myofilaments to calcium [93]. Almost 5% of the infused dose of Levosimendan is converted by the small intestine to its active metabolite OR-1896, which has an elimination half-life of 75 to 80 h, allowing cardiovascular effects to persist up to 7 to 9 days after the discontinuation of the initial infusion [94]. 

The mechanism of action of levosimendan is complex as it involves both an active long-lived metabolite and interactions with more than one molecular target within the cardiovascular system. The primary mechanisms involve the interaction with calcium-saturated myofilaments of cardiomyocytes, stabilizing their binding with Ca+ ions [95], and promoting contractile force while leaving diastolic function unimpaired and oxygen consumption unaltered [96]. Vasodilatation is mediated by its activity in opening ATP-sensitive K+ channels of vascular smooth muscle cells, leading to their hyperpolarization and consequent relaxation [97]. The highly selective inhibition of the phosphodiesterase PDE-3 isoform increases intracellular cAMP concentration [98] with positive inotropy and lusitropy in cardiomyocytes. Activity in mitochondrial adenosine triphosphate synthesis determines cardioprotection and possible neuronal protection during ischemic insults; indeed, a significant decrease in circulating brain natriuretic peptide (BNP) levels has been observed, and this decrease has been positively correlated with improvements in clinical outcomes at 6 months in patients with congestive heart failure [99].

#### Cerebral Oxygenation Effects

Due to its neuroprotective properties, levosimendan has been studied as a potentially beneficial drug for preventing secondary brain injury resulting from hypotension, hypoxia, hypercapnia and hypocapnia. Various animal experimental models have demonstrated improvements in both cardiac arrest scenarios, where levosimendan was able to increase coronary perfusion pressure and elevated regional brain oxygen saturation [100], and in TBI cases, where the drug led to dose-dependent reductions in total and secondary tissue injury at the 72 h time point [101].

Limited evidence is available regarding studies investigating the direct role of levosimendan in improving cerebral oxygenation. Garcia-Bardon et al. conducted a study using an animal experimental model to examine the impact of levosimendan on hemodynamic parameters, cerebral perfusion and oxygenation in juvenile male pigs following the return of spontaneous circulation after cardiac arrest induced by ventricular fibrillation [102]. The animals that received levosimendan exhibited higher brain PbtO_2_ levels without any modifications in cardiac output, preload, afterload, arterial blood pressure or changes in cerebral microcirculation. The study also demonstrated that Levosimendan could potentially enhance cerebral oxygen levels after global cerebral ischemia-reperfusion injury, independent of its effects on cardiac output or brain tissue perfusion.

### 6.2. Vasopressin

Vasopressin, also known as antidiuretic hormone or arginine vasopressin, is a peptide hormone primarily synthesized in the hypothalamus and stored in the posterior pituitary gland [103]. Structurally, it is a nonapeptide with a cyclic six-amino acid ring and a three-amino acid tail. The synthetic forms of vasopressin are known as “terlipressin” or “desmopressin” [104,105].

Physiologically, vasopressin plays a crucial role in maintaining osmotic balance and blood pressure. Its primary action is the conservation of body water by reducing the excretion of water in urine. This action is mediated through V2 receptors, which activate the aquaporin-2 water channels in the kidney nephron [106]. Moreover, vasopressin has been implicated in a variety of central nervous system functions, including the regulation of circadian rhythms, thermoregulation and the modulation of stress responses, as well as contributing to social behavior, memory and learning.

In addition to these functions, vasopressin has a significant role in cardiovascular homeostasis. It acts on V1 receptors located on vascular smooth muscle to induce vasoconstriction, thereby increasing systemic vascular resistance and arterial blood pressure. This vasoconstrictive effect is particularly important in situations of hypovolemia or hemorrhage, where vasopressin release is upregulated as a compensatory mechanism, and which explains its therapeutic use in vasodilatory shock. Unlike other vasopressor agents, vasopressin possesses the ability to dilate certain vascular beds in addition to its vasoconstrictive effects [107].

#### Cerebral Oxygenation Effects

Animal studies have demonstrated that vasopressin can influence cerebral blood flow and brain oxygenation [108]. For instance, in pig models undergoing cardiopulmonary resuscitation, vasopressin was found to enhance cerebral blood flow and oxygenation more effectively than epinephrine [109]. Similarly, these beneficial effects compared with epinephrine have also been found to ameliorate cerebral microcirculation, with greater post-resuscitation microvascular flows and a greater number of cortical micro-vessels perfused [110]. However, these findings are not consistent across all studies, with some reporting contradictory results [111]. Additionally, in models of hemorrhagic shock, vasopressin did not show significant advantages over epinephrine in restoring hemodynamics, CPP or in maintaining brain oxygenation [112]. 

Contrasting these positive outcomes, a prospective, single-blind study in humans under anesthesia found that while arginine vasopressin prevented hypotension, it adversely affected cerebral tissue oxygen saturation, causing increased cerebral desaturation compared to a control group receiving normal saline [113]. Similarly, a recent animal study investigating terlipressin effectiveness in restoring cerebral oxygenation in TBI and hemorrhagic shock models found no improvement in cerebral perfusion pressure or cerebral oximetry, despite hemodynamic recovery across all study groups [114].

### 6.3. Angiotensin II

Angiotensin II, a key element in the renin–angiotensin system, is an octapeptide hormone that plays a vital role as the principal mediator of this system in the regulation of blood pressure and fluid equilibrium [115]. It influences cerebral blood flow through its vasoconstrictive effects on cerebral arteries [116], thereby affecting cerebral perfusion pressure. Despite this, our search for studies specifically investigating the impact of angiotensin II on cerebral oxygenation did not yield significant results.

In addition, angiotensin II modulates the blood–brain barrier’s permeability [117], and has been implicated in inflammatory responses within the central nervous system [118]. These actions are particularly relevant in conditions like TBI, stroke and SAH, where the dysregulation of cerebral hemodynamics and inflammation are key pathological features. For this reason, angiotensin II could potentially worsen brain injuries [119], and medications that inhibit angiotensin II type 1 receptors, like sartans, might offer neuroprotective, neurorestorative and anti-inflammatory benefits in brain-injured patients [120,121].

## 7. Discussion

According to the latest TBI management guidelines [3], it is advised to maintain a systolic blood pressure higher than 100 mmHg in patients 50 to 69 years old and higher than 110 mmHg in patients younger than 49 or over 70 years old. Additionally, a target CPP range of 60–70 mmHg is recommended to improve chances of survival and achieve favorable outcomes. In cases of SAH, it is advisable to raise blood pressure in patients experiencing delayed cerebral ischemia (DCI), unless their baseline blood pressure is already high or their cardiac condition prevents it, maintaining a MAP above 90 mm Hg [122,123].

These guidelines are grounded in equation (1), previously outlined, which states that CPP depends on MAP and ICP. However, when considering the impact of CPP on CBF, the effects of administered drugs, which may induce alterations in tissue perfusion and, consequently, oxygenation, are not always taken into consideration. This can have detrimental effects in situations where even slight alterations in tissue perfusion or oxygenation can lead to secondary damage to the brain tissue. A proposal suggests that cardiac output plays a crucial role in enhancing brain oxygenation, as the impact on the latter appears to be directly proportional to changes in the former [9]. 

Despite having similar hemodynamic parameters, different vasopressors can evoke varying effects on cerebral oxygenation. For example, a reduction in SctO_2_, albeit minimal, resulted after the administration of α-adrenergic drugs (phenylephrine and noradrenaline) but not after administering β-adrenergic drugs (epinephrine) to treat anesthesia-induced hypotension [124]. An explanation could be that the impact on α receptors in resistance arterioles can potentially lead to decreased oxygenation compared to β-mediated vasodilation.

In animal models, dopamine and norepinephrine exhibited distinct impacts, as dopamine infusion led to an increase in local CBF and moderate decreases in glucose utilization, while norepinephrine administration did not significantly alter CBF and glucose utilization in most brain regions [125]. In TBI patients, dopamine has demonstrated a more pronounced and variable effect on cerebral oxygen consumption than norepinephrine, significantly increasing from baseline [64]. Furthermore, dopamine administration has been associated with a significant elevation in lactate and pyruvate levels. Similarly, when used to augment CPP in TBI patients, norepinephrine appears to have a more predictable effect in increasing the mean flow velocity of the middle cerebral artery (MCA) evaluated with transcranial Doppler, compared to dopamine [126]. Similarly, levosimendan has demonstrated its potential to augment cerebral oxygen levels after global cerebral ischemia-reperfusion injury [102], independent of its impact on cardiac output or brain tissue perfusion. This pharmaceutical agent functions as a phosphodiesterase inhibitor without known effects on adrenoreceptors. 

The BBB is known to contain MAO [22,23], which may limit or prevent the systemic vasoconstrictive effects of noradrenaline from affecting cerebral circulation. However, emerging studies indicate that high doses of noradrenaline or damage to the BBB can be associated with cerebral vasoconstriction [127,128]. For example, during DCI in SAH patients, hypertensive therapy with norepinephrine has been shown to deteriorate the neurological status progressively with dose escalation [129]. These observations open new avenues for future research, such as in the assessment of cerebrovascular resistance and the analysis of transcranial Doppler (TCD) curves as markers for BBB disruption, as indicators of noradrenaline-induced cerebral vasoconstriction, or to identify noradrenaline dosage thresholds that may lead to cerebral vasoconstriction. 

Finally, the correlation between brain tissue oxygenation and metabolic effects remains inadequately understood, as already described by Johnston [69], and which can explain the lack of strong evidence when evaluating the metabolic effectiveness of different medications. Various reasons can be used to explain the challenges in comparing tissue oxygenation and metabolism. First, a significant metabolic decrease may manifest solely under extremely low levels of oxygen tension [130]. Secondly, an ischemic burden, if present, may not diminish even with an elevation in tissue oxygen levels. This could be due to factors such as mitochondrial dysfunction [131] or minimal burden from the outset. The metabolic effects may align with the alterations in brain tissue oxygenation, but the timeframe required for the changes in one to impact the other remains unknown. For example, in the cited study by Lang et al. [71] evaluating the effects of manipulating arterial pressure with norepinephrine in TBI patients, the authors obtained a captivating correlation between PtbO_2_ and the CPP. Indeed, this relationship exhibited an ellipsoidal pattern and was characterized by a time delay ranging from 20 s to 3 min for changes in tissue oxygen pressure to follow shifts in arterial blood pressure. These results are similar to the findings reported in Mutoh’s figures regarding the increase in dobutamine levels and the subsequent rise in regional brain oxygen saturation [80]. These patterns show a plateau phase in PbtO2, as also evidenced in the study by Kiening et al. [60]. However, the Lang study observed a broader plateau of PbtO_2_ during CPP increase, which can be attributed to various factors such as (1) variances in baseline PbtO_2_ levels, (2) the specific site of injury being monitored and (3) the use of different drugs. This observation may corroborate the notion that the effects on cerebral oxygenation encompass mechanisms that remain not completely understood.

This review primarily focuses on the effects of vasopressors and inotropes; however, other often-used medications, such as clonidine and dexmedetomidine [132], as well as beta-blockers, exert activity on the adrenoceptors and should be considered as potential confounding factors to achieve a comprehensive understanding. In the end, all of this evidence contributes to defining a significant aspect of vasopressor therapy in managing patients with TBI and SAH: the importance of multimodal neuro monitoring (MMM) [133], as the management of an adequate CPP cannot disregard the evaluation of PbtO_2_ [9], and the need for a comprehensive and deeper understanding of how these drugs function and exert their effects within the cerebral context.

## 8. Conclusions

In the clinical management of a patient suffering from either traumatic brain injury (TBI) or subarachnoid hemorrhage (SAH), preserving an appropriate cerebral perfusion pressure (CPP) is a pivotal treatment strategy. However, the selection of varying vasopressors or inotropes can exert differential effects on the cerebrovascular system and cerebral oxygenation, which, in turn, may influence patient outcomes in unique ways. 

A profound comprehension of the sympathetic nervous system’s activity and receptor functionality within cerebral structures is required to appreciate these impacts and their potential clinical ramifications. Limited research has specifically scrutinized the influence of distinct vasopressors and inotropes on cerebral oxygenation in patients with brain injuries. Evaluating the effectiveness of vasopressors and inotropes merely based on CPP fails to provide an exhaustive understanding of the actual effects at the cerebral tissue level, necessitating a comprehensive monitoring strategy encompassing multiple factors.

## Data Availability

Not applicable.

## Abbreviations

5-HT	5-Hydroxytryptamine
ABI	Acute Brain Injury
ACA	Anterior Cerebral Artery
AVDO_2_	Arterial Venous Differences of Oxygen
BBB	Blood–Brain Barrier
BNP	Brain Natriuretic Peptide
cAMP	Cyclic Adenosine Monophosphate
CBF	Cerebral Blood Flow
CMR	Cerebral Metabolic Ratio
CMRO_2_	Cerebral Metabolic Oxygen Consumption
CO	Cardiac Output
CPP	Cerebral Perfusion Pressure
CVR	Cerebral Vascular Resistance
DAG	Diacylglycerol
DCI	Delayed Cerebral Ischemia
GCS	Glasgow Coma Scale
ICA	Internal Carotid Artery
ICH	Intracerebral Hemorrhage
ICP	Intracranial Pressure
ICU	Intensive Care Unit
IP3	Inositol Triphosphate
MAO	Monoamine Oxidases
MAP	Mean Arterial Pressure
MCA	Middle Cerebral Artery
MMM	Multimodal Neuro Monitoring
NIRS	Near-Infrared Spectroscopy
OCI	Oxygen Carbohydrate Index
OEF	Oxygen Extraction Fraction
OG	Otic Ganglion
PDE	Phosphodiesterase
PbtO_2_	Brain Tissue Oxygenation
PCA	Posterior Cerebral Artery
SAH	Subarachnoid Hemorrhage
SCG	Superior Cervical Ganglion
SctO_2_	Cerebral Tissue Oxygen Saturation
SjvO_2_	Jugular Venous Oxygen Saturation
SPG	Sphenopalatine Ganglion
TBI	Traumatic Brain Injury
TCD	Transcranial Doppler
TG	Trigeminal Ganglion
VA	Vertebral Artery
